# Osteonecrosis of femoral head: Treatment by core decompression and vascular pedicle grafting

**DOI:** 10.4103/0019-5413.45320

**Published:** 2009

**Authors:** Sudhir Babhulkar

**Affiliations:** Former Professor and Head of Orthopaedics, Indira Gandhi Medical College, Nagpur; Director, Sushrut Hospital, Research Centre and Postgraduate Institute of Orthopaedics, Nagpur, India

**Keywords:** Muscle pedicle bone graft, osteonecrosis of femoral head, vascularized pedicle graft

## Abstract

**Background:**

Femoral head-preserving core decompression and bone grafting have shown excellent result in preventing collapse. The use of vascularized grafts have shown better clinical results. The vascular pedicle bone graft is an easy to perform operation and does not require special equipment. We analyzed and report a series of patients of osteonecrosis of femoral head treated by core decompression and vascular pedicle grafting of part of iliac crest based on deep circumflex iliac vessels.

**Materials and Methods:**

The article comprises of the retrospective study of 31 patients of osteonecrosis of femoral head in stage II and III treated with core decompression and vascular pedicle grafting by using part of iliac crest with deep circumflex iliac vessels from January 1990 to December 2005. The young patients with a mean age 32 years (18–52 years) with a minimum follow-up of five years were included for analysis. Sixteen patients had osteonecrosis following alcohol abuse, 12 patients following corticosteroid consumption, 3 patients had idiopathic osteonecrosis. Nine patients were stage IIB, and 22 patients were stage IIIC according to ARCO's system. The core decompression and vascular pedicle grafting was performed by anterior approach by using part of iliac crest with deep circumflex iliac vessels.

**Results:**

Digital subtraction arteriography performed in 9 patients at the end of 12 weeks showed the patency of deep circumflex artery in all cases, and bone scan performed in 6 other patients showed high uptake in the grafted area of the femoral head proving the efficacy of the operative procedure. Out of 31 patients, only one patient progressed to collapse and total joint replacement was advised. At the final follow up period of 5–8 years, Harris Hip Score improved mean ± SD of 28.2 ± 6.4 (*p* < 0.05). Forty-eight percent of patients had an improvement in Harris Hip Score of more that 28 points.

**Conclusion:**

The core decompression and vascular pedicle grafting reduces the intraosseous tension to achieve early revascularization of ischemic femoral head. The high percentage of marrow and osteogenic cells survive within a vascularized pedicle graft, which helps in early vascularization and we have been able to achieve good outcome.

## INTRODUCTION

Osteonecrosis of femoral head is a disabling condition frequently seen in association with many disorders like corticosteroids consumption, alcohol abuse, hemoglobinopathy (sickle cell disease and coagulopathies), certain renal, hepatic and skin disorders and commonly affects young patients around the age 20–40 years.^1^,^2^ It is often characterized by relentless progression despite treatment, resulting in to subchondral fracture, collapse and painful disabling arthrosis.^3^ Hence, it is essential to diagnose and treat the patients of osteonecrosis early to prevent the disintegration and collapse of femoral head. The true prevalence of the disease is difficult to ascertain. Advanced osteonecrosis with secondary osteoarthritis is reported in 5% to 18% of total patients undergoing total hip replacement in the US.^4^–^6^

The average age of patients in a large series was found to be between 34 and 38 years, with only 20% of patients being older than 50 years of age.^5^,^6^ Osteonecrosis is usually associated with one or more risk factors, approximately two-thirds of this is related to alcohol abuse and corticosteroid intake. The remaining third are associated with diverse conditions like sickle cell haemoglobinopathy, storage disorders like Gaucher's disease, pregnancy, coagulopathies and decompression sickness, organ transplant recipients, inflammatory bowel disease, and lupus erythematosus. Patients with history of pain in the groin, radiating pain in thigh and symptoms mimicking prolapsed intervertebral disc should be grouped as high index of suspicion kept under constant follow up in this group of disorders. The reported incidence of bilaterality ranges from 6 to 72%.^7^–^9^ Despite a high incidence of bilaterality, only about 15% of patients report with bilateral symptoms on initial presentation.^9^ The difficulty in estimating the prevalence of osteonecrosis arises because the condition is asymptomatic in the early stages. The term “Silent Hip” is applied to the asymptomatic hip in patients who present for the management of the contralateral painful hip.^7^

The natural history of osteonecrosis of femoral head before the development of crescent sign or before the collapse of the femoral head has never been well defined. The possibility of progression to collapse is thought to increase after the development of an abnormality that can be seen on plain radiograph and such possibility and the course of collapse may be highly variable and unknown.^6^ It is generally agreed that symptomatic, radiographically abnormal hip will progress to collapse of the femoral head when treated nonoperatively.^6^ To avoid these complications and to avoid early replacement arthroplasty in young group of patients, many operative procedures to salvage the femoral head are in vogue.

Head-preserving operation of core decompression and various types of bone grafting certainly gives excellent results in early stage of osteonecrosis. Despite the many reports on the utility of various operative procedures, no single method has uniformly demonstrated the arrest of disease or prevention of collapse of the femoral head effectively. The vascularized fibular grafting is associated with better clinical and radiographic results than is nonvascularized fibular grafting in precollapse hips.^6^ However, the successful use of free vascularized bone grafts requires a meticulous process of procuring the vascular fibula with microanastomosis to the recipient site. The microsurgical procedures require specialized training, equipment and expertise. The vascular pedicle bone graft by using part of iliac crest based on deep circumflex iliac vessel described in this article is easy to perform and does not require any special equipment or technique, and still has the advantages of the vascularized bone graft. We analyzed and report a series of patients of osteonecrosis of femoral head treated by core decompression and vascular pedicle grafting of part of iliac crest based on deep circumflex iliac vessels.

## MATERIALS AND METHODS

This retrospective study comprises of 31 patients of osteonecrosis femoral head treated with core decompression and vascular pedicle bone grafting by using deep circumflex iliac vessels based part of iliac crest, from Jan 1990 to Dec 2005 with a minimum follow up of five years and average follow up of 8 years. All these patients had important clinical signs such as loss of internal rotation of hip and positive axis deviation test, which is highly suggestive of osteonecrosis of femoral head. The axis deviation test is positive whenever there is sectoral involvement of femoral head. All were investigated by X-rays, bone scan and magnetic resonance imaging (MRI) to ascertain the diagnosis and early diagnosis and staging^10^–^14^ and detect the involvement of contraleteral asymptomatic hip. The international classification system proposed by the Association Research Circulation Osseous (ARCO) has been used [Table 1] which includes radiographs, computed tomography (CT), bone scans, and MRI.^15^ The diagnostic criteria of avascular necrosis was considered as established if any of the following were found:

1. Pathognomonic radiographic changes (collapse of the femoral head, anterolateral sequestration, crescent sign).^16^,^17^
2. A double line on T2-weighted MRI.^18^
3. Increased uptake surrounding a photopenic area of bone scan (cold in hot).^18^
4. Positive finding on bone biopsy – showing empty lacunae involving multiple adjacent trabeculae.^15^

**Table 1:** ARCO's classification

Stage	0	1		2	3	4
Findings	All present techniques normal or non diagnostic	X-ray and CT are normal. At least one of the below is positive		No crescent sign: X-ray abnormal: Sclerosis, lysis, focal porosis	Crescent sign on the X-ray and/ or flattening of articular surface of femoral head	Osteoarthritis joint space narrowing, acetabular changes, joint destruction
Techniques	X-ray	Scintigraph		X-ray, CT	X-ray, CT only	X-ray only
	CT	MRI		Scintigraph	*Quantitate on X-ray
	Scintigraph	*Quantitate on		MRI		
	MRI	MRI		*Quantitate MRI and X-ray		
Subclassification	No	Medical	Central	Lateral		No
		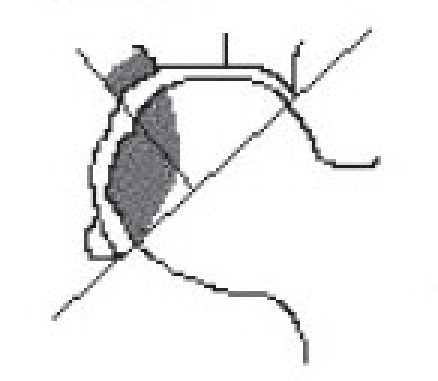	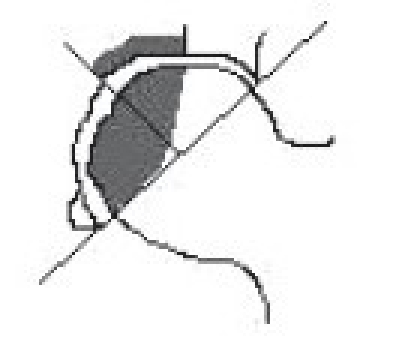	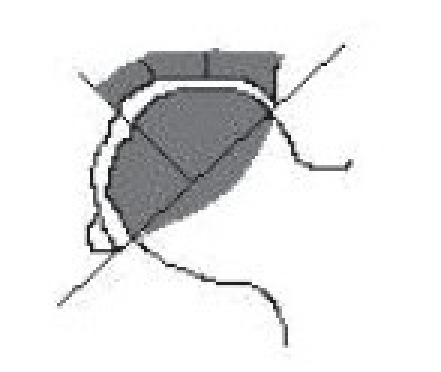		
Quantitation	No	Quantitation				No
		% Area Length % Surface collapse			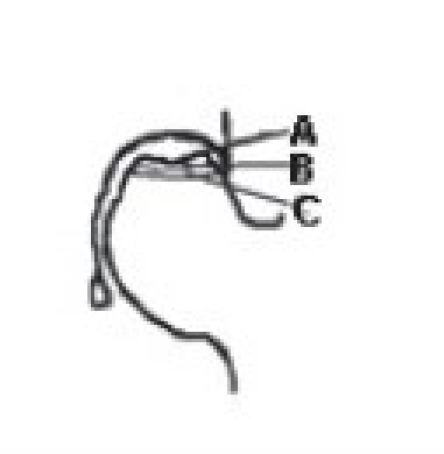
		Involvement of crescent dome depression			
		Minimal A < 15% A < 15% A < 2 mm			
		Moderate B 15–30% B 15–30% B 2–4 mm			
		Extensive C > 30% C >30% C > 4 mm			

Whenever bone scan was negative in such high-risk group of patients with strong clinical suspicion, the patients were subjected to sequential MRI, especially on the contralateral side. All patients were young with a mean age of 32 years (range: 18–52 years) with 26 males and 5 females. Hematological investigations for hepatic, renal function and coagulopathy to assess the hepatorenal function were carried out in each individual. Nine patients were stage IIB, and 22 patients were stage IIIC according to ARCO's system. Sixteen patients had osteonecrosis following alcohol abuse, 12 patients following corticosteroid consumption, 3 patients had idiopathic osteonecrosis. Seven patients had bilateral involvement, where only one hip was operated with vascular pedicle graft and the other hip with early involvement (Grade I) were treated with core decompression and free fibular graft [Table 2]. Once the surgery of vascular pedicle graft was planned, preoperative angiography was performed on each patient to document the presence of deep circumflex iliac artery. Follow up by clinical assessment and radiological examination was done every 3 months for 1 year, every 6 months for the next 5 years and then yearly follow up thereafter. Initially in 15 patients, digital subtraction arteriography (*n* = 9) was performed at the end of 12 weeks, which showed the patency of deep circumflex artery, and bone scan (*n* = 6) showed high uptake in the grafted area of the femoral head proving the efficacy of the operative procedure. However, this investigation was not performed routinely after proving the efficacy in first 15 cases. *Harris hip score system*^19^ was used for assessment of the clinical results.

**Table 2:** Stages of osteonecrosis and the age and sex of patients

Stage of osteonecrosis	No. of patients	No. of patient and sex of the patients	Age of patient			
			
			16–20	21–30	31–40	41 and above
II	9 patients	7 males	-	3	1	3
		2 female	1	1	-	-
III	22 patients	19 males	2	3	11	3
		3 females	-	1	2	-
Total	31 patients	26 males	3	8	14	6
		05 females

### Operative procedure

**Figure 1:**
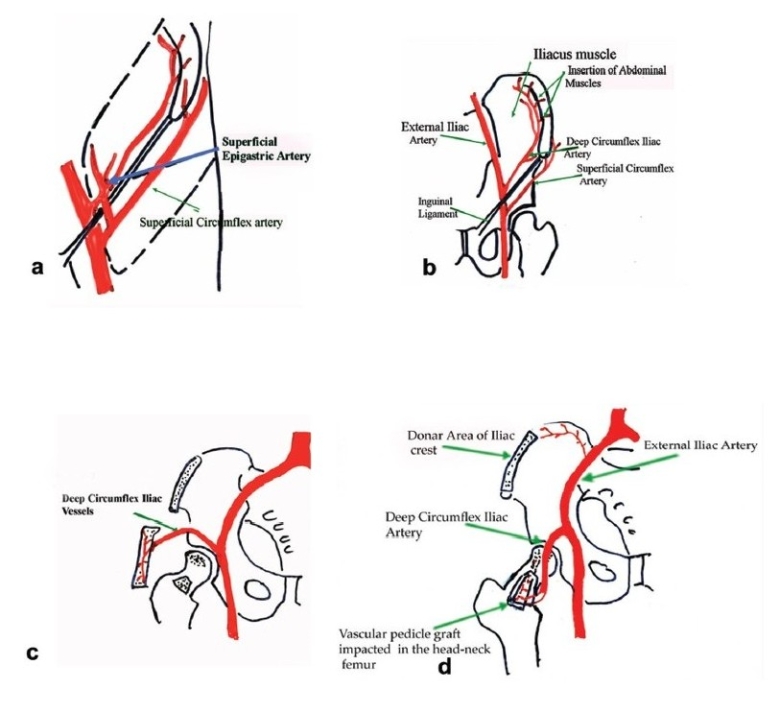
(a, b) Line diagram (L. D.) showing anatomical landmarks and diagrammatic representation of the vascular pedicle of deep circumflex iliac artery in the vicinity of hip joint and iliac bone. (c) L. D. showing isolation of deep circumflex iliac vascular pedicle along with part of iliac bone. (d) L. D. showing implantation of vascular pedicle graft of deep circumflex iliac artery with part of iliac crest into the head and neck of femur.

Harvesting the vascular iliac crest graft requires careful isolation of deep circumflex iliac vessel from its origin.^25^,^26^ Patient after necessary (usually spinal) anesthesia were put in supine position. A curvilinear incision was taken at the mid-inguinal region extending along the inguinal ligament from the point of femoral pulsations proximally to the uppermost convexity of iliac crest and distally was turned anterolaterally up to the sub-trochanteric region. Subsequently, the inguinal ligament was properly exposed and retracted upwards to expose the femoral artery, which is traced above the inguinal ligament where it becomes external iliac artery. The origin of inferior gastric artery on the medial side of external iliac artery, is identified and opposite to it is the origin of deep circumflex iliac artery which can be easily identified laterally, [Figure 1a]. Once the deep circumflex iliac artery is identified, which is constantly found, it is traced upwards and laterally toward anterior superior iliac spine and iliac crest [Figure 1b]. The iliac crest was freed from inner lip by erasing three abdominal muscles till we just reach the top of the iliacus muscle. Similarly the iliac crest was freed by stripping the tensor fascia and gluteus medius on external surface. This isolation of iliac crest graft was best done by subperiosteal separation of muscles on either side of lip of the ilium without disturbing the vascular supply. The desired size of free vascularized iliac bone is now marked on the external surface and osteotomy of iliac crest was performed with sharp osteotome or pneumatic saw. Properly planned free iliac crest graft along with vascular pedicle of deep circumflex iliac artery is now ready for transfer [Figure 1b–d].

Now the inferior part of curvilinear incision is extended from medial side to anterolaterally up to the subtrochanteric region. Subsequently, the hip joint was exposed by retracting gluteus medius laterally and by erasing vastus lateralis from anterior portion of subtrochanteric femur, which will expose the hip joint capsule anteriorly. The hip capsule was cut in T-shaped manner and the femoral head was exposed after confirming the necrotic lesion under image intensifier. The window was made at the junction of femoral head and neck by drilling initially by 4.5 mm extending upwards through the ischemic area. Now the entire core decompression procedure was done. Gradually, the size of drilling was increased and entire ischemic sectoral involvement is drilled usually up to 12–14 mm, then the created core tract, cavity and ischemic segment of femoral head was curetted. If there was a depressed subchondral bone, it was raised up by using an impactor under image control and collapsed part of the femoral head was raised up, and the subchondral area and part of the cavity was filled with few pieces of cancellous bone removed from the iliac crest procured while harvesting the vascular pedicle. The trough and cavity was now ready for receiving the vascular iliac crest graft [Figure 1c, Figure 1d]. Now the graft was tunneled through the intermuscular plane between rectus femoris and pectineus muscle and is swung in to the trochanteric region and is introduced in to the trough created in the femoral head and confirmed under the image intensifier [Figure 1d]. The graft was secured by taking the stitch through the hole prepared by drilling in the neck and graft.

Postoperatively, the limb was kept in 20° abduction and 30° flexion and 10° of internal rotation to avoid tension on the vascular pedicle. The patients were mobilized by allowing free movements of flexion extension and adduction abduction after 15 days in bed, and after 4–6 weeks, patients were mobilized out of bed on non-weight bearing crutch walking with encouragement to perform all movements. The patients were allowed partial weight bearing after 10 weeks and full weight bearing after 14–16 weeks depending upon the incorporation of graft.

## RESULTS

Of the 31 femoral heads, almost all patients had relief of pain with improvement in the range of movements at the end of 10–12 weeks. Six patients (grade III) had residual low intensity pain for a period of 24 weeks. Eight patients had painless limp for a period of 16–18 weeks. Four patients of Grade III had restriction of flexion beyond 100–110° almost till the last follow up at 5 years. Only one hip from stage III progressed to further collapse and got deformed, but without any progression to arthrosis and total hip replacement was advised at 6 years of follow up. Another one patient had superficial infection at the operative site, and the wound responded to dressing and short course of antibiotics within 15 days of surgery. No other complications occurred in any other patient. Nine patients of stage II completely improved without any further deterioration with complete relief of pain. The postoperative bone scan (n=6) and digital subtraction arteriogram (n=9) performed after 12 weeks, showed hundred percent patency of the vascular pedicle and viability of the graft. The clinical and radiological results were available for all these patients.

The mean ± SD improvement in Harris Hip Score [Table 3] at 5–8 years follow up was 28.6 ± 6.4. The difference in the pre- and postoperative score across the whole sample was significant (*P* < 0.05). When analysed separately for patients with stage II and stage III disease, there was no significant difference in the Harris Hip Score, suggesting that at least in the present sample, the stage of the disease had no effect on improvement seen after surgery. Seventy percent of patients had improvement in Harris Hip Score of > 25 points, while 48% had improvement in the score of more than 28 points.

**Table 3:** Showing preoperative and postoperative total Harris hip scores stagewise

Sr. No.	Patient's initials	Stage	Preoperative score	Postoperative score
1	PC	IIIC	49	88
2	SK	IIIC	57	85
3	SR	IIB	57	82
4	GK	IIIC	48	76
5	SG	IIIC	48	76
6	MD	IIIC	47	74
7	LK	IIC	50	88
8	AD	IIIC	48	81
9	SS	IIC	55	90
10	SC	IIC	50	71
11	KJ	IIIC	67	82
12	GK	IIIC	50	84
13	SS	IIIC	65	82
14	RR	IIIC	49	83
15	GH	IIIC	56	90
16	MP	IIIC	48	73
17	GP	IIIC	50	69
18	NL	IIIC	56	79
19	SK	IIIC	49	85
20	AN	IIIC	57	84
21	PD	IIIC	56	89
22	RL	IIIC	48	73
23	MD	IIIC	57	84
24	YK	IIB	50	73
25	RN	IIC	55	79
26	MS	IIIC	48	69
27	AS	IIIC	49	73
28	RC	IIC	56	91
29	SK	IIIC	48	75
30	LD	IIB	50	88
31	BM	IIIC	55	86

Pre-operative score: 40–49 = *n* = 11Postoperative score: 65–49 = *n* = 950–60 = *n* = 1870–80 = *n* = 961 and above = *n* = 281 and above = *n* = 13

**Figure 2:**
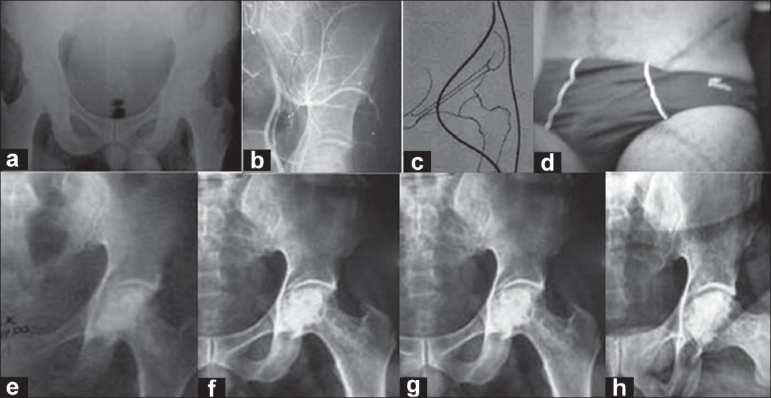
A young male (case no. 7) age 35 years had bilateral osteonecrosis femoral head (steroid-induced osteonecrosis). (a) Preoperative anteroposterior X-ray of left hip shows stage III osteonecrosis. (b) Preoperative angiography showing the presence of deep circumflex iliac pedicle. (c) Line diagram shows proposed surgical incision for exploration of vascular pedicle graft and its implantation into the head and neck femur. (d) Clinical photograph showing operative scar. (e,f) Postoperative anteroposterior X-rays at three years showing good revascularization with preservation of joint space. (g,h) Postoperative anteroposterior X-rays at 5 and 10 years showing good revascularization, preserved joint space with no arthritic changes and no deformation.

**Figure 3:**
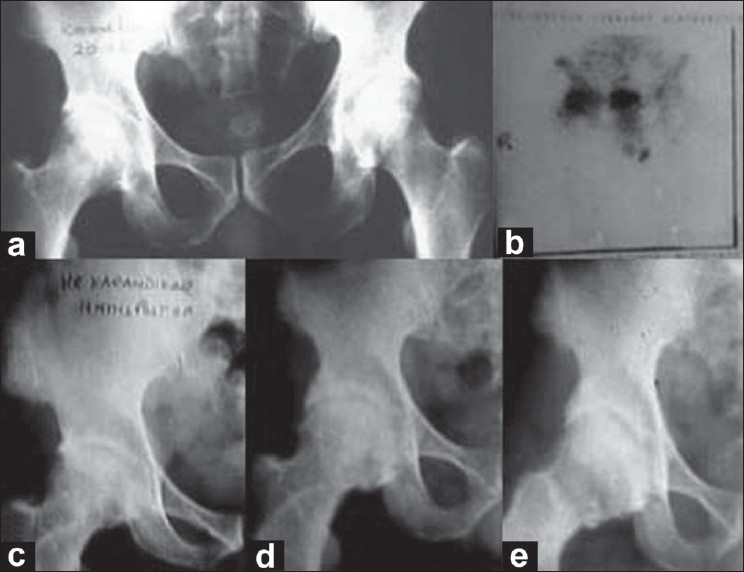
Pre-operative anteroposterior (a) X-ray of the pelvis of young male patient shows osteonecrosis of the femoral head. (b) Preoperative radionuclide bone scan showing osteonecrosis of right femoral head. (c) Immediate postoperative X-rays after vascular pedicle bone grafting. (d) Postoperative X-ray at 14 months showing good revascularization of right femoral head. (e) Postoperative X-rays at 15 years showing good revascularization of right femoral head without any collapse or arthritic changes.

**Figure 4:**
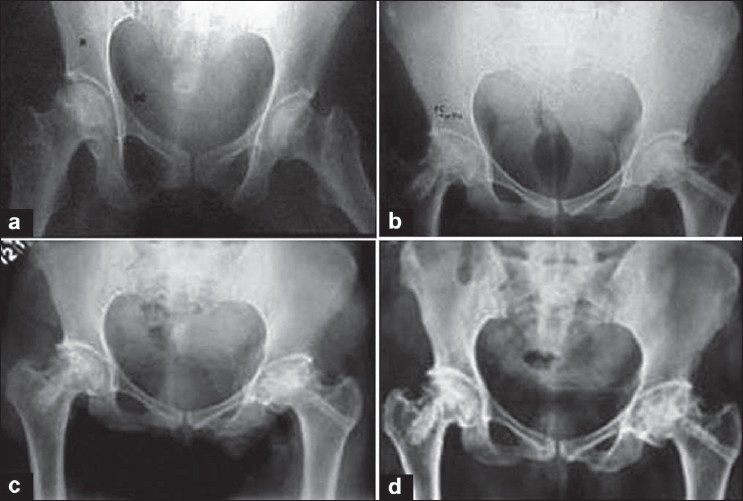
(a) Preoperative X-rays of the pelvis with both hips of a young lady shows osteonecrosis of both femoral heads. (b) Postoperative X-rays of the same patient at 2 years, where vascular pedicle graft from deep circumflex iliac artery (right side) and fibular bone graft (left side) was done. (c) Postoperative X-ray of the same patient at 5 years showing good vascularization of the right femoral head and postoperative. (d) X-ray at 10 years showing good revascularization of the femoral heads without any collapse or arthritic changes.

Out of 31 operated patients, 9 patients of stage II recovered completely and no collapse occurred till the last follow up of more than 8 years. Out of 22 patients of stage III, 12 patients had excellent results and had no symptoms of pain or restricted movements and all of them returned to their respective jobs [Figure 2]. Out of remaining ten patients, six patients had residual pain for about 6 months, but these patients also had complete relief from pain and returned to their employment [Figure 3, Figure 4]. Amongst the remaining four patients, in one patient, the head progressively collapsed and developed deformity causing disturbing pain, to whom total hip replacement was advised and the other three patients accepted the limitation of flexion. The sphericity of the femoral head was maintained in all the patients at 5-year follow up, except for the slight flattening and dome depression that occurred in two patients.

## DISCUSSION

Need to treat ischemia of femoral head is becoming more common since many cases are detected in early stages in young patients. One must consider the possibility of osteonecrosis if individual has pain in the vicinity of hip that had history of chronic alcoholism, corticosteroid consumption, associated disease like sickle cell, Gaucher's, Gout etc.^8^,^31^,^32^ Early diagnosis prior to the appearance of radiological changes is crucial in the treatment of ischemic necrosis. Its diagnosis is based on clinical examination and by bone scan, CT, and MRI as osteonecrosis is the response to the vascular impairment of the bone marrow circulation. X-ray examination is of limited value in early stage of but has importance in staging since it helps in planning the treatment and the prognosis. The X-ray become positive late in the condition after the process of repair has started. The ischemic death of bony and marrow tissues occur in osteonecrosis. Different imaging modalities provide different information on the mineralized and non-mineralized component of the bone. Though bone scan is also important but MRI has dramatically improved the diagnosis of osteonecrosis, about 30–70% cases of femoral head osteonecrosis, the other hip is affected in due course of time. Hence, it is necessary to exclude early involvement of the contralateral hip, which is asymptomatic by either bone scan or MRI. MRI is the most accurate imaging modality for the diagnosis of osteonecrosis of femoral head, especially in the early stages when there are only bone marrow changes. Characteristic MRI signal alterations in the anterosuperior portion of the femoral head surrounded by a band of low signal intensity on T1- and T2-weighted images represent the diagnostic criteria of osteonecrosis on MRI.^18^ The occurrence of a double-line sign on the T2-weighted image represents a pathognomonic sign, but its absence does not eliminate the diagnosis of osteonecrosis.^33^

Marcus *et al.*,^34^ Steinberg *et al.*,^13^,^35^ Ficat and Arlet,^36^,^37^ and ARCO's classification^15^ are the various staging systems in vogue for osteonecrosis, but with inherent problems of poor reliability.

The Association Research Circulation Osseous (ARCO) has proposed a new international classification system including radiographs, computed tomography (CT), bone scans, and MRI [Table 1]. This classification system incorporates the lesion size and the lesion location. Quantitation (% area involvement of femoral head, length of crescent sign, % surface collapse, and dome depression) and location of the lesion (medial, central or lateral) represent important prognostic factors. This ARCO's classification has been proposed as the preferred system for the future, which is used in this study.^15^

Core decompression offers the opportunity to study histological changes of early bone ischemia. It also achieves reduction in the symptoms of pre-collapse stage of ischemic necrosis because of reduction of pressure in the compartment.

Barring exceptional circumstances, there is hardly any role of conservative treatment of osteonecrosis of femoral head and surgery is inevitable. Steinberg *et al.*^13^ reported that progression occurred in 92% of 48 hips that had undergone nonoperative management. While observing the patients with protected weight bearing, more than 85% patients had collapse of femoral head at 2 years when symptomatic hips with stage I and II were left untreated.^17^,^20^ More studies have shown that nonoperative treatment yields poor results. The only condition for which the protected weight bearing might be effective is a type A lesion i.e. involvement of medial aspect of femoral head. No drugs have been useful and specific in the treatment of osteonecrosis, though recently the use of Alendronate has been advised.

Once diagnosed, it is desirable to subject the patient to early surgical intervention. Rationale for the treatment of osteonecrosis of femoral head requires a lot of consideration.^5^,^20^ Prime importance should be given to the age of the patient, whether both hips are affected, etiology of the associated diseases, functional demands of the patients, and the stage of the disease when the patient presents for treatment. Only core decompression may relieve the pain^21^ but is not useful for revascularization of femoral head; hence, core decompression should always be supplemented by one of the procedures of bone grafting. Core decompression is an effective treatment in the preradiological and precollapse stage of avascular necrosis of the femoral head,^8^,^36^,^37^ especially if coupled with bone grafting. Jones^39^ analyzed nine studies and showed that in 218 of 369 patients (59%), where core decompression was performed in the precollapse stage, failed to prevent the progressive collapse. Steinberg *et al.*^13^ concluded that core decompression provided more predictable pain relief and changed the indications for arthroplasty more consistently than conservative management. However, only core decompression should be avoided, and it must be coupled with bone grafting in the tract of core to avoid iatrogenic fractures.^40^ Despite many reports on salvage procedure, no method has clearly demonstrated the arrest of disease before subchondral fracture or slow down of the progression of collapse of femoral head and arthrosis.^6^

The use of a nonvascularized bone graft, as originally described by Phemister, has had variable success in the treatment of osteonecrosis. Marcus *et al.*^34^ reported satisfactory clinical results in seven of eleven hips at the time of short-term follow up (range: 2–4 years). The other workers concluded that Phemister bone-grafting technique is not effective once collapse has occurred. Boettcher *et al.*^24^ reported success in 27 (71%) of 38 hips 6 years after nonvascularized tibial strut grafting. However, a longer-term evaluation (performed at a mean of 14 years postoperatively) that included the original 38 hips in the study by Boettcher *et al.* found that only 16 (29%) of 56 hips still had a good result.^41^ Once the crescent sign appears without collapse, it is desirable to couple the bone grafting procedure in addition to the core decompression, preferably vascular or muscle pedicle grafting, to achieve early revascularization. Vascularized pedicle graft by using part of iliac crest with deep circumflex iliac vessels is more advantageous since high percentage of marrow and osteogenic cells survive within a living graft, which helps for early vascularization.^25^–^29^ However, muscle pedicle graft using tensor fascia lata graft is very easy and is commonly performed whenever both hips need simultaneous surgery. Muscle pedicle graft using quadratus femoris (Meyer's procedure)^23^ was also propagated in the treatment of osteonecrosis, but it did not achieve satisfactory early revascularization and went into disrepute since the results were not encouraging, though Meyer's^23^ reported the success rate of 57% and Baksi^30^ reported 93% good results. Use of tensor fascia lata graft is commonly advocated when bilateral hips are involved and surgery is performed in the single sitting.

The study by Plakseychuk *et al.*^27^,^28^ on free vascular fibular grafting showed better clinical results and prevention of radiographic signs of progression and collapse of the femoral head more frequently than does nonvascularized fibular grafting. A marked difference with regard to signs of radiographic progression and collapse was noted between the A and B subgroups in the precollapse groups (Stages I and II). The potential disadvantages of vascularized fibular grafting are a longer operation time, need of microvascular technique, leaves a longer operative scar, and is associated with more donor site morbidity such as ankle instability, toe-clawing, subtrochanteric fracture, and heterotopic ossification. To achieve early vascularization^25^–^26^ vascular pedicle grafting using deep circumflex iliac vessel with iliac crest is very useful [Figures 2–4]. This procedure though easy is technically demanding and time consuming. Preoperative femoral angiography is necessary to confirm the presence of deep circumflex iliac artery pedicle [Figure 2B]. The vascularized grafting provided a significant benefit for hips in Stages IB, IIA, and IIB.^27^

The rationale of this procedure of vascularized pedicle bone grafting is based on the following three points:

1. Decompression of the femoral head, which acts as compartment syndrome following increased intraosseous pressure and interrupts the circulation that is thought to contribute to the disease
2. Excision of the necrotic tissue, which inhibits revascularization of the head
3. Filling of the defect that is created after core with vascular pedicle, an osteoinductive cancellous graft, which is a viable and supports the subchondral surface and enhances the revascularization process

It does not require advanced training of microsurgical technique nor any special equipment and can be performed by any average orthopaedic surgeon. Morbidity of the donor site is minimal and operative time required is comparable to a total hip arthroplasty, and all problems and obstacles associated with vascularized fibular graft are avoided by this technique.^41^

Head-preserving operation of core decompression and vascular pedicle grafting certainly gives excellent results in stages II and III. The prognosis of stages II and III is fairly good, whereas in stage IV, it is not satisfactory since about 1/3 of the stage IV group are likely to progress further and may require total hip joint replacement or resurfacing operations.^42^,^43^ Prosthetic replacement is frequently an unappealing option for patients who have osteonecrosis because many are young and the etiological factors associated with the disease are also associated with complications after total hip arthroplasty, hemiarthroplasty, and surface replacement.^6^ Though at our institute, many patients of sickle cell disease with osteonecrosis are studied and treated, this procedure of vascular pedicle grafting was not performed on any such patient because of the possibility and high prevalence of thrombo-embolism in the vascular tree in this disease. Out of 103 patients treated by Urbanaiak *et al.*,^6^ by free vascular fibular grafting, total hip replacement was performed in 34% cases in stages II and III within 5 years. There was survivorship and the probability of conversion within 5 years to THR rate of 11% in stage II and 23% survival for stage III. In the study of Shin Yoon Kim,^41^ the rate of conversion to total hip replacement was 13% (three of 23 hips) in the vascularized graft group and 22% (five of 23 hips) in the nonvascularized graft group, in comparison only one patient out of 31 pts of vascular pedicle grafting was advised total hip replacement in the present series at the end of 6 years. The hips treated with vascular pedicle grafting seemed to have less dome depression of the femoral head and the retention of sphericity, probably because of more rapid revascularization and increased osteoinductive potential of the vascularized graft. It has been observed that there is an early failure of total hip replacement in osteonecrosis than in age-matched patients with other diagnosis because of abnormal remodeling of bones and subsidence of prosthesis because of poor quality of proximal femoral bone.^40^ Other contributory factors for failure are – ongoing systemic disease, defects in mineral metabolism, use of steroids, high level of activity in young patients and increased body weight. Hence, we prefer to delay or eliminate the need for hip replacement by performing head-preserving surgeries, of which core decompression and vascular pedicle grafting are a choice of surgery.^40^

## CONCLUSION

If osteonecrosis of femoral head is diagnosed early, head-preserving operation of core decompression and vascular pedicle bone grafting yield good to excellent results in stages II and III. Out of 31 patients, only one patient progressed to collapse, and surgery of joint replacement was advised.
